# 1–10-100: Unifying goals to mobilize global action on antimicrobial resistance

**DOI:** 10.1186/s12992-024-01070-8

**Published:** 2024-08-27

**Authors:** Susan Rogers Van Katwyk, Mathieu J. P. Poirier, Sujith J. Chandy, Kim Faure, Caitlin Fisher, Guillaume Lhermie, Arshnee Moodley, Satyajit Sarkar, Masika Sophie, Kayla Strong, Isaac Weldon, Steven J. Hoffman

**Affiliations:** 1 Global Strategy Lab, Dahdaleh Institute for Global Health Research, York University, 4700 Keele Street, Dahdaleh Building 2120, Toronto, M3J 2S5 Canada; 2 School of Global Health, Faculty of Health, York University, Toronto, M3J 2S5 Canada; 3 International Centre for Antimicrobial Resistance Solutions (ICARS), Copenhagen, Denmark; 4 Global Antibiotic R&D Partnership, 1202 Geneva, Switzerland; 5 School of the Arts, Media, Performance, and Design, York University, Toronto, M3J 2S5 Canada; 6 School of Public Policy, University of Calgary, Calgary, T2P 1H9 Canada; 7 Faculty of Veterinary Medicine, University of Calgary, Calgary, T2N 4Z6 Canada; 8 One Health at UCalgary, University of Calgary, Calgary, T2N 1N4 Canada; 9 Department of Veterinary and Animal Sciences, University of Copenhagen, 1870 Frederiksberg, Denmark; 10 Animal and Human Health Programme, International Livestock Research Institute, Nairobi, Kenya; 11 Department of Policy & Economic Research, International Vaccine Institute, Seoul, 08826 Korea; 12 World Federation for Animals, Boston, USA; 13 Centre for Law, Medicine, and Life Sciences, Faculty of Law, University of Cambridge, Cambridge, CB3 9DZ UK; 14 Osgoode Hall Law School, York University, Toronto, M3J 1P3 Canada

**Keywords:** Antimicrobial resistance, Unifying goals, Global governance, One health, Sustainable development goals

## Abstract

The Bellagio Group for Accelerating AMR Action met in April 2024 to develop the ambitious but achievable 1–10-100 unifying goals to galvanize global policy change and investments for antimicrobial resistance mitigation: 1 Health; 10 million lives saved; and 100% sustainable access to effective antimicrobials. High profile political goals such as the Paris Agreement’s objective to keep global warming well below 2° Celsius compared to pre-industrial levels, UNAIDS’ 90–90-90 goal, and the Sustainable Development Goals challenge global norms, direct attention towards relevant activities, and serve an energizing function to motivate action over an extended period of time. The 1–10-100 unifying goals propose to unite the world through a One Health approach to safeguard human health, animal welfare, agrifood systems, and the environment from the emergence and spread of drug-resistant microbes and infections; save over 10 million lives by 2040 through concerted efforts to prevent and appropriately treat infections while preserving the vital systems and services that depend on sustained antimicrobial effectiveness; and commit to ensuring that antimicrobials are available and affordable for all, used prudently, and secured for the future through innovation. Compared to existing technical targets, these unifying goals offer advantages of focusing on prevention, encouraging multisectoral action and collaboration, promoting health equity, recognizing the need for innovation, and integrating with Sustainable Development Goals. By committing to 1 Health, 10 million lives saved, and 100% sustainable access to effective antimicrobials, we can protect lives and livelihoods today and safeguard options for tomorrow.

## Main text

The rapid global increase in drug-resistant infections threatens to undermine decades of progress on global health and development, exacerbating inequalities between the Global North and Global South [1]. The human burden of antimicrobial resistance (AMR) – which was associated with 4.95 million deaths in 2019 – falls disproportionately on low- and middle-income countries (LMICs) and particularly on children under five [2]. AMR is also expected to reduce global animal production by up to 7.5%, resulting in economic losses up to one trillion dollars, increasing the cost of food [3], and undermining decades of progress in food security and nutrition worldwide [4]. These interconnections between people, animals, agrifood systems, and our shared environment means that a collaborative and multisectoral “One Health” approach is fundamental to addressing AMR [5].

As world leaders convene in New York to chart a path forward on AMR in a United Nations General Assembly High-Level Meeting this September, it is imperative that they set out an ambitious vision that drives progress for the coming decades [6, 7]. Goal setting is an inherently political challenge: goals are tools of global governance that act as vehicles for global norms, direct attention and effort towards relevant activities, and serve an energizing function to motivate action over an extended period of time [8]. High profile goals such as the Millennium Development Goals (MDGs) [9] and subsequent Sustainable Development Goals (SDGs) [10], the Paris Agreement’s objective to keep global warming well below 2° Celsius compared to pre-industrial levels [11], and UNAIDS’ 90–90-90 goal [12] make concrete a shared vision for the future and rally support for action to address a global challenge.

*The Bellagio Group for Accelerating AMR Action* met from April 8th to 12th 2024 to identify unifying global goals for AMR which could, 1) unite technical perspectives across all countries and sectors into a memorable concept that is easily communicated, 2) act as a barometer of global progress by providing a framework into which an action-oriented roadmap can be crafted with concrete and sector-specific targets, and 3) be useable by heads of government and ministers when communicating with citizens and journalists about the importance of action on AMR while providing identifiable moments of success [7].

**Fig. 1:**
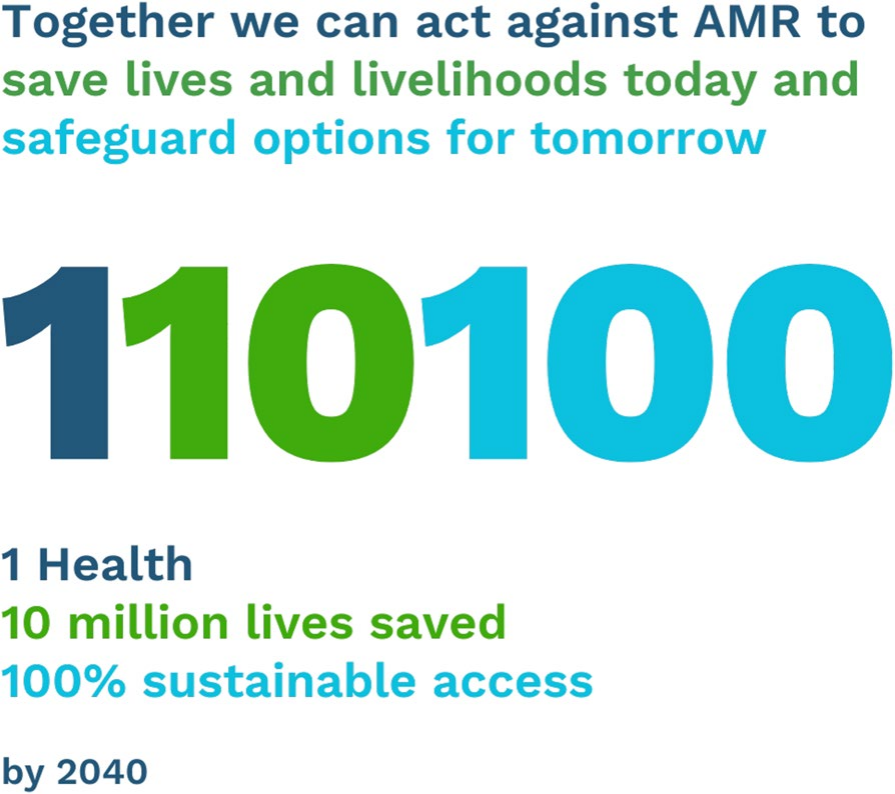
The 1–10-100 unifying goals for AMR action by 2040

Our 1–10-100 goals (Fig. 1) put forward an ambitious but achievable vision for AMR action by 2040. These goals will build momentum to ensure that we can save lives and livelihoods today and safeguard options for tomorrow. Its three priorities are:

### 1 Health

We must unite the world through a One Health approach to safeguard human health, animal welfare, agrifood systems, and the environment from the emergence and spread of drug-resistant microbes and infections. Progress towards this goal can be measured through more secure livelihoods (e.g., percent reduction in pathogen introduction and spread between farms; percent reduction in people forced into extreme poverty by AMR) and sustained biodiversity (e.g., percent of land and ocean designated as protected).

### 10 million lives saved

We can save over 10 million lives by 2040 through concerted efforts to prevent and appropriately treat infections while preserving the vital systems and services that depend on sustained antimicrobial effectiveness. Progress towards this goal can be measured through enhanced vaccination programs (e.g., percent vaccination coverage), improved water, sanitation, and hygiene (WASH) infrastructure (e.g., percent access to clean water and sanitation), and improvements to infection prevention and control (IPC) measures (e.g., percent compliance to IPC and biosecurity measures), thereby preventing loss in economic productivity (e.g., percent GDP loss averted).

### 100% sustainable access

We must commit to ensuring that antimicrobials are available and affordable for all, used prudently, and secured for the future through innovation. Progress can be measured through greater access (e.g., percent access to quality-assured antimicrobials, diagnostics, and health services), enhanced conservation (e.g., percent of antimicrobials used that meet treatment guidelines; percent reduction of non-veterinary and non-phytosanitary use of antimicrobials in the agrifood system), and innovations (e.g., number of new antimicrobials, vaccines, diagnostics, and social innovations).

The Global Leaders Group on AMR (GLG), an independent advisory group of world leaders and AMR experts, recently proposed global targets of reducing bacterial AMR deaths by 10%, ensuring ACCESS group antibiotics^1^ comprise over 80% of human antibiotic consumption, reducing antimicrobials used in the agrifood system by 30–50%, and eliminating the unnecessary use of medically important antimicrobials for human medicine in animals and agrifood systems [14]. The 2024 Lancet Series on Sustainable Access to Antibiotics has similarly proposed a 10% reduction in mortality from AMR, a 20% reduction in inappropriate human antibiotic use, and a 30% reduction in inappropriate animal antibiotic use [15]. We welcome the growing consensus on the need for global targets to assess progress in addressing AMR and point to five advantages of using the 1–10-100 goals to structure these more technical objectives.

First, the 1–10-100 goals are prevention-focused, combining an evidence-based approach to disease prevention and sustainable husbandry practices through investments in effective interventions such as vaccinations, IPC and biosecurity practices, and WASH infrastructure to save 10 million lives by 2040. These ambitious goals are possible because some of the most cost-effective and impactful interventions to sustain antimicrobial effectiveness are the same interventions that are urgently needed to protect lives and livelihoods from preventable infectious diseases [16].

Second, they are equity-promoting, as expanding access to effective antimicrobials while investing in prevention-focused interventions will most benefit LMICs, newborns and children, and marginalized communities, where the burden of infectious disease is highest, access to effective antimicrobials is the lowest, and health and food systems are least prepared to respond [2]. However, taking steps towards universal access to effective antimicrobials will require difficult negotiations of international trade law, where countries often do not utilize TRIPS flexibilities like compulsory licensing for fear of the economic consequences [17].

Third, our proposal recognizes the need to address AMR through multisectoral action and collaboration by putting One Health first and fully integrating the health of humans, animals, plants, agrifood systems, and the environment in the achievement of every goal. This approach seeks to avoid the scapegoating of animal husbandry and agricultural sectors as bearing disproportionate blame for the acceleration of AMR in human health, and instead highlight the shared benefits of protecting livelihoods that would otherwise be lost due to resistant infections among plants and animals, ultimately resulting in considerable costs to communities and national economies [3].

Fourth, it recognizes the need for innovation to secure a future where effective antimicrobials and alternative treatments are available to all when needed, including the need for new equity-promoting diagnostics, vaccines, and social innovations.

Finally, it integrates with existing SDGs across One Health sectors related to universal health coverage, access to WASH, food security, and maternal and child health, thereby leveraging investments to effectively address AMR within a shorter timeframe.

The upcoming United Nations General Assembly High-Level Meeting on AMR represents a landmark opportunity to adopt the 1–10-100 goals. We recognize that achieving 100% sustainable access to antimicrobials is an aspirational goal, similar to the SDG’s vision of ending poverty in all its forms everywhere, serving as a unifying objective for the global AMR community. Saving 10 million lives is feasible if political leaders embrace a One Health approach, integrating whole-of-government and a whole-of-society strategies. However, if international declarations continue to focus on highly technical targets, the absence of an ambitious yet achievable political vision for AMR could delay global action, at the cost of many lives and livelihoods.

Communicating the risk posed by AMR to political leaders and the public has always been challenging, especially when framed in technical terms. To effectively address AMR, we need a robust communications strategy that brands and popularizes the unifying goals, while fostering direct political engagement at both national and international levels, with active support from the private sector, academia, and civil society. The United Nations’ endorsement of the 1–10-100 goals would bring much-needed legitimacy, and monitoring of progress in saving 10 million lives and achieving the technical targets within each goal could be an important role played by an Independent Panel on Evidence for Action against AMR.

The lack of a unifying global vision of success for action on AMR has meant that past efforts have not achieved the necessary level of ambition and coordination to create meaningful change [6]. We believe that the 1–10-100 goals put forward a new vision for AMR that is easy to communicate to the public, that is centred on tangible One Health actions, and has the potential to galvanize policy change and investments in AMR. By committing to 1 Health, 10 million lives saved, and 100% sustainable access to effective antimicrobials, we can protect lives and livelihoods today and safeguard options for tomorrow.

## Data Availability

No datasets were generated or analysed during the current study.

## Footnotes

1 ACCESS group antibiotics are recommended for the empiric treatment of common infections due to their narrow spectrum of activity, fewer side effects, and lower risk of developing resistance with widespread use [13].
